# Supplementary material for: A fault-intrusion-tolerant system and deadline-aware algorithm for scheduling scientific workflow in the cloud
Source: PeerJ Comput Sci. 2021 Nov 2;7:e747. doi: 10.7717/peerj-cs.747 (PMC8576554; doi:10.7717/peerj-cs.747)
Supplement: Supplemental Information 6 [file peerj-cs-07-747-s006.zip › Myproject1.1/docs/org/cloudbus/cloudsim/CloudletSchedulerTimeShared.html]

CloudletSchedulerTimeShared


JavaScript is disabled on your browser.


- Overview
- Package
- Class
- Use
- Tree
- Deprecated
- Index
- Help

- Prev Class
- Next Class

- Frames
- No Frames

- All Classes

- Summary:
- Nested |
- Field |
- Constr |
- Method

- Detail:
- Field |
- Constr |
- Method


org.cloudbus.cloudsim

## Class CloudletSchedulerTimeShared

- java.lang.Object
- - org.cloudbus.cloudsim.CloudletScheduler
  - - org.cloudbus.cloudsim.CloudletSchedulerTimeShared

- Direct Known Subclasses:
  :   CloudletSchedulerDynamicWorkload

  ---

    

  ```
  public class CloudletSchedulerTimeShared
  extends CloudletScheduler
  ```

  CloudletSchedulerTimeShared implements a policy of scheduling performed by a virtual machine.
  Cloudlets execute time-shared in VM.

  Since:
  :   CloudSim Toolkit 1.0

- - ### Field Summary

    Fields

    | Modifier and Type | Field and Description |
    | --- | --- |
    | `protected int` | `currentCPUs` The current cp us. |
  - ### Constructor Summary

    Constructors

    Constructor and Description

    `CloudletSchedulerTimeShared()`

    Creates a new CloudletSchedulerTimeShared object.
  - ### Method Summary

    Methods

    | Modifier and Type | Method and Description |
    | --- | --- |
    | `Cloudlet` | `cloudletCancel(int cloudletId)` Cancels execution of a cloudlet. |
    | `void` | `cloudletFinish(ResCloudlet rcl)` Processes a finished cloudlet. |
    | `boolean` | `cloudletPause(int cloudletId)` Pauses execution of a cloudlet. |
    | `double` | `cloudletResume(int cloudletId)` Resumes execution of a paused cloudlet. |
    | `double` | `cloudletSubmit(Cloudlet cloudlet)` Receives an cloudlet to be executed in the VM managed by this scheduler. |
    | `double` | `cloudletSubmit(Cloudlet cloudlet, double fileTransferTime)` Receives an cloudlet to be executed in the VM managed by this scheduler. |
    | `protected double` | `getCapacity(java.util.List<java.lang.Double> mipsShare)` Gets the capacity. |
    | `protected <T extends ResCloudlet>  java.util.List<T>` | `getCloudletExecList()` Gets the cloudlet exec list. |
    | `protected <T extends ResCloudlet>  java.util.List<T>` | `getCloudletFinishedList()` Gets the cloudlet finished list. |
    | `protected <T extends ResCloudlet>  java.util.List<T>` | `getCloudletPausedList()` Gets the cloudlet paused list. |
    | `int` | `getCloudletStatus(int cloudletId)` Gets the status of a cloudlet. |
    | `java.util.List<java.lang.Double>` | `getCurrentRequestedMips()` Gets the current requested mips. |
    | `double` | `getCurrentRequestedUtilizationOfBw()` Gets the current requested bw. |
    | `double` | `getCurrentRequestedUtilizationOfRam()` Gets the current requested ram. |
    | `Cloudlet` | `getNextFinishedCloudlet()` Returns the next cloudlet in the finished list, $null if this list is empty. |
    | `double` | `getTotalCurrentAllocatedMipsForCloudlet(ResCloudlet rcl, double time)` Gets the total current allocated mips for cloudlet. |
    | `double` | `getTotalCurrentAvailableMipsForCloudlet(ResCloudlet rcl, java.util.List<java.lang.Double> mipsShare)` Gets the total current mips for the Cloudlet. |
    | `double` | `getTotalCurrentRequestedMipsForCloudlet(ResCloudlet rcl, double time)` Gets the total current requested mips for cloudlet. |
    | `double` | `getTotalUtilizationOfCpu(double time)` Get utilization created by all cloudlets. |
    | `boolean` | `isFinishedCloudlets()` Informs about completion of some cloudlet in the VM managed by this scheduler. |
    | `Cloudlet` | `migrateCloudlet()` Returns one cloudlet to migrate to another vm. |
    | `int` | `runningCloudlets()` Returns the number of cloudlets runnning in the virtual machine. |
    | `protected <T extends ResCloudlet>  void` | `setCloudletExecList(java.util.List<T> cloudletExecList)` Sets the cloudlet exec list. |
    | `protected <T extends ResCloudlet>  void` | `setCloudletFinishedList(java.util.List<T> cloudletFinishedList)` Sets the cloudlet finished list. |
    | `protected <T extends ResCloudlet>  void` | `setCloudletPausedList(java.util.List<T> cloudletPausedList)` Sets the cloudlet paused list. |
    | `double` | `updateVmProcessing(double currentTime, java.util.List<java.lang.Double> mipsShare)` Updates the processing of cloudlets running under management of this scheduler. |

    - ### Methods inherited from class org.cloudbus.cloudsim.CloudletScheduler

      `getCurrentMipsShare, getPreviousTime, setCurrentMipsShare, setPreviousTime`
    - ### Methods inherited from class java.lang.Object

      `clone, equals, finalize, getClass, hashCode, notify, notifyAll, toString, wait, wait, wait`

- - ### Field Detail


    - #### currentCPUs

      ```
      protected int currentCPUs
      ```

      The current cp us.
  - ### Constructor Detail


    - #### CloudletSchedulerTimeShared

      ```
      public CloudletSchedulerTimeShared()
      ```

      Creates a new CloudletSchedulerTimeShared object. This method must be invoked before starting
      the actual simulation.
  - ### Method Detail


    - #### updateVmProcessing

      ```
      public double updateVmProcessing(double currentTime,
                              java.util.List<java.lang.Double> mipsShare)
      ```

      Updates the processing of cloudlets running under management of this scheduler.

      **Specified by:**
      :   `updateVmProcessing` in class `CloudletScheduler`

      Parameters:
      :   `currentTime` - current simulation time
      :   `mipsShare` - array with MIPS share of each processor available to the scheduler

      Returns:
      :   time predicted completion time of the earliest finishing cloudlet, or 0 if there is
          no next events


    - #### getCapacity

      ```
      protected double getCapacity(java.util.List<java.lang.Double> mipsShare)
      ```

      Gets the capacity.

      Parameters:
      :   `mipsShare` - the mips share

      Returns:
      :   the capacity


    - #### cloudletCancel

      ```
      public Cloudlet cloudletCancel(int cloudletId)
      ```

      Cancels execution of a cloudlet.

      **Specified by:**
      :   `cloudletCancel` in class `CloudletScheduler`

      Parameters:
      :   `cloudletId` - ID of the cloudlet being cancealed

      Returns:
      :   the canceled cloudlet, $null if not found


    - #### cloudletPause

      ```
      public boolean cloudletPause(int cloudletId)
      ```

      Pauses execution of a cloudlet.

      **Specified by:**
      :   `cloudletPause` in class `CloudletScheduler`

      Parameters:
      :   `cloudletId` - ID of the cloudlet being paused

      Returns:
      :   $true if cloudlet paused, $false otherwise


    - #### cloudletFinish

      ```
      public void cloudletFinish(ResCloudlet rcl)
      ```

      Processes a finished cloudlet.

      **Specified by:**
      :   `cloudletFinish` in class `CloudletScheduler`

      Parameters:
      :   `rcl` - finished cloudlet


    - #### cloudletResume

      ```
      public double cloudletResume(int cloudletId)
      ```

      Resumes execution of a paused cloudlet.

      **Specified by:**
      :   `cloudletResume` in class `CloudletScheduler`

      Parameters:
      :   `cloudletId` - ID of the cloudlet being resumed

      Returns:
      :   expected finish time of the cloudlet, 0.0 if queued


    - #### cloudletSubmit

      ```
      public double cloudletSubmit(Cloudlet cloudlet,
                          double fileTransferTime)
      ```

      Receives an cloudlet to be executed in the VM managed by this scheduler.

      **Specified by:**
      :   `cloudletSubmit` in class `CloudletScheduler`

      Parameters:
      :   `cloudlet` - the submited cloudlet
      :   `fileTransferTime` - time required to move the required files from the SAN to the VM

      Returns:
      :   expected finish time of this cloudlet


    - #### cloudletSubmit

      ```
      public double cloudletSubmit(Cloudlet cloudlet)
      ```

      **Description copied from class: `CloudletScheduler`**

      Receives an cloudlet to be executed in the VM managed by this scheduler.

      **Specified by:**
      :   `cloudletSubmit` in class `CloudletScheduler`

      Parameters:
      :   `cloudlet` - the submited cloudlet

      Returns:
      :   expected finish time of this cloudlet, or 0 if it is in a waiting queue


    - #### getCloudletStatus

      ```
      public int getCloudletStatus(int cloudletId)
      ```

      Gets the status of a cloudlet.

      **Specified by:**
      :   `getCloudletStatus` in class `CloudletScheduler`

      Parameters:
      :   `cloudletId` - ID of the cloudlet

      Returns:
      :   status of the cloudlet, -1 if cloudlet not found


    - #### getTotalUtilizationOfCpu

      ```
      public double getTotalUtilizationOfCpu(double time)
      ```

      Get utilization created by all cloudlets.

      **Specified by:**
      :   `getTotalUtilizationOfCpu` in class `CloudletScheduler`

      Parameters:
      :   `time` - the time

      Returns:
      :   total utilization


    - #### isFinishedCloudlets

      ```
      public boolean isFinishedCloudlets()
      ```

      Informs about completion of some cloudlet in the VM managed by this scheduler.

      **Specified by:**
      :   `isFinishedCloudlets` in class `CloudletScheduler`

      Returns:
      :   $true if there is at least one finished cloudlet; $false otherwise


    - #### getNextFinishedCloudlet

      ```
      public Cloudlet getNextFinishedCloudlet()
      ```

      Returns the next cloudlet in the finished list, $null if this list is empty.

      **Specified by:**
      :   `getNextFinishedCloudlet` in class `CloudletScheduler`

      Returns:
      :   a finished cloudlet


    - #### runningCloudlets

      ```
      public int runningCloudlets()
      ```

      Returns the number of cloudlets runnning in the virtual machine.

      **Specified by:**
      :   `runningCloudlets` in class `CloudletScheduler`

      Returns:
      :   number of cloudlets runnning


    - #### migrateCloudlet

      ```
      public Cloudlet migrateCloudlet()
      ```

      Returns one cloudlet to migrate to another vm.

      **Specified by:**
      :   `migrateCloudlet` in class `CloudletScheduler`

      Returns:
      :   one running cloudlet


    - #### getCloudletExecList

      ```
      protected <T extends ResCloudlet> java.util.List<T> getCloudletExecList()
      ```

      Gets the cloudlet exec list.

      Type Parameters:
      :   `T` - the generic type

      Returns:
      :   the cloudlet exec list


    - #### setCloudletExecList

      ```
      protected <T extends ResCloudlet> void setCloudletExecList(java.util.List<T> cloudletExecList)
      ```

      Sets the cloudlet exec list.

      Type Parameters:
      :   `T` - the generic type

      Parameters:
      :   `cloudletExecList` - the new cloudlet exec list


    - #### getCloudletPausedList

      ```
      protected <T extends ResCloudlet> java.util.List<T> getCloudletPausedList()
      ```

      Gets the cloudlet paused list.

      Type Parameters:
      :   `T` - the generic type

      Returns:
      :   the cloudlet paused list


    - #### setCloudletPausedList

      ```
      protected <T extends ResCloudlet> void setCloudletPausedList(java.util.List<T> cloudletPausedList)
      ```

      Sets the cloudlet paused list.

      Type Parameters:
      :   `T` - the generic type

      Parameters:
      :   `cloudletPausedList` - the new cloudlet paused list


    - #### getCloudletFinishedList

      ```
      protected <T extends ResCloudlet> java.util.List<T> getCloudletFinishedList()
      ```

      Gets the cloudlet finished list.

      Type Parameters:
      :   `T` - the generic type

      Returns:
      :   the cloudlet finished list


    - #### setCloudletFinishedList

      ```
      protected <T extends ResCloudlet> void setCloudletFinishedList(java.util.List<T> cloudletFinishedList)
      ```

      Sets the cloudlet finished list.

      Type Parameters:
      :   `T` - the generic type

      Parameters:
      :   `cloudletFinishedList` - the new cloudlet finished list


    - #### getCurrentRequestedMips

      ```
      public java.util.List<java.lang.Double> getCurrentRequestedMips()
      ```

      **Description copied from class: `CloudletScheduler`**

      Gets the current requested mips.

      **Specified by:**
      :   `getCurrentRequestedMips` in class `CloudletScheduler`

      Returns:
      :   the current mips


    - #### getTotalCurrentAvailableMipsForCloudlet

      ```
      public double getTotalCurrentAvailableMipsForCloudlet(ResCloudlet rcl,
                                                   java.util.List<java.lang.Double> mipsShare)
      ```

      **Description copied from class: `CloudletScheduler`**

      Gets the total current mips for the Cloudlet.

      **Specified by:**
      :   `getTotalCurrentAvailableMipsForCloudlet` in class `CloudletScheduler`

      Parameters:
      :   `rcl` - the rcl
      :   `mipsShare` - the mips share

      Returns:
      :   the total current mips


    - #### getTotalCurrentAllocatedMipsForCloudlet

      ```
      public double getTotalCurrentAllocatedMipsForCloudlet(ResCloudlet rcl,
                                                   double time)
      ```

      **Description copied from class: `CloudletScheduler`**

      Gets the total current allocated mips for cloudlet.

      **Specified by:**
      :   `getTotalCurrentAllocatedMipsForCloudlet` in class `CloudletScheduler`

      Parameters:
      :   `rcl` - the rcl
      :   `time` - the time

      Returns:
      :   the total current allocated mips for cloudlet


    - #### getTotalCurrentRequestedMipsForCloudlet

      ```
      public double getTotalCurrentRequestedMipsForCloudlet(ResCloudlet rcl,
                                                   double time)
      ```

      **Description copied from class: `CloudletScheduler`**

      Gets the total current requested mips for cloudlet.

      **Specified by:**
      :   `getTotalCurrentRequestedMipsForCloudlet` in class `CloudletScheduler`

      Parameters:
      :   `rcl` - the rcl
      :   `time` - the time

      Returns:
      :   the total current requested mips for cloudlet


    - #### getCurrentRequestedUtilizationOfRam

      ```
      public double getCurrentRequestedUtilizationOfRam()
      ```

      **Description copied from class: `CloudletScheduler`**

      Gets the current requested ram.

      **Specified by:**
      :   `getCurrentRequestedUtilizationOfRam` in class `CloudletScheduler`

      Returns:
      :   the current requested ram


    - #### getCurrentRequestedUtilizationOfBw

      ```
      public double getCurrentRequestedUtilizationOfBw()
      ```

      **Description copied from class: `CloudletScheduler`**

      Gets the current requested bw.

      **Specified by:**
      :   `getCurrentRequestedUtilizationOfBw` in class `CloudletScheduler`

      Returns:
      :   the current requested bw


- Overview
- Package
- Class
- Use
- Tree
- Deprecated
- Index
- Help

- Prev Class
- Next Class

- Frames
- No Frames

- All Classes

- Summary:
- Nested |
- Field |
- Constr |
- Method

- Detail:
- Field |
- Constr |
- Method
